# Effect of therapy on Quantiferon-Plus response in patients with active and latent tuberculosis infection

**DOI:** 10.1038/s41598-018-33825-w

**Published:** 2018-10-23

**Authors:** Elisa Petruccioli, Teresa Chiacchio, Valentina Vanini, Gilda Cuzzi, Luigi Ruffo Codecasa, Maurizio Ferrarese, Vincenzo Schininà, Fabrizio Palmieri, Giuseppe Ippolito, Delia Goletti

**Affiliations:** 1 Translational Research Unit National Institute for Infectious Diseases, Lazzaro Spallanzani IRCCS, Rome, Italy; 2 Regional TB Reference Centre, Istituto Villa Marelli, Ospedale Niguarda, Milano, Italy; 3 Clinical Department National Institute for Infectious Diseases Lazzaro Spallanzani IRCCS, Rome, Italy; 4 Scientific Direction, National Institute for Infectious Diseases Lazzaro Spallanzani IRCCS, Rome, Italy

## Abstract

Lack of biomarkers for treatment monitoring is listed among the main requirements for next generation assays, as identified globally among tuberculosis (TB) researchers. In this study, we evaluated in a low TB endemic country such as Italy, the effect of preventive therapy on the results obtained in the QuantiFERON TB Plus (QFT-Plus), in a cohort of subjects with latent TB infection (LTBI) and active TB. We found that TB therapy significantly decreased IFN-γ values and number of responders to TB1- and TB2- peptides stimulation in both LTBI and active TB patients. Stratifying LTBI subjects according to the type of preventive TB therapy used, we found that INH treatment but not INH and RIF significantly decreased IFN-γ production. Stratifying the active TB patients according the microbiological status, we found that TB therapy significantly decreased IFN-γ response to antigen present in QFT-Plus test in patients with clinical diagnosis compared to those with a microbiological diagnosis. In conclusions, we demonstrated that TB therapy decreases IFN-γ level in response to antigen present in QFT-Plus test in LTBI and active TB patients. Future studies are needed to better characterize Mtb-specifc response as a potential marker for monitoring TB therapy and preventive treatment effects.

## Introduction

Tuberculosis (TB) has been responsible for 10.4 million cases and 1.7 million deaths in 2017, representing one of the major public health problem^1^. Considering that latent TB infection (LTBI) affect one-fourth of the world’s population and that it may progress to active disease in about 3–15% of the cases^2,3^, it is obvious that to diagnose and treat the reservoir of the disease is one of the main goals to control and eliminate the TB epidemic^4^. Currently, we need to improve tools for monitoring TB therapy and preventive TB therapy efficacy^5^. In particular, in case of extra-pulmonary TB, being impossible to detect *M. tuberculosis* (Mtb) in a “relative easy to take” sample as sputum^6^, it is not possible to correlate the sputum result to the clinical outcome. It is even more difficult in LTBI subjects, in which is not possible to isolate Mtb despite its presence. The discovery of new biomarker assays to diagnose Mtb infection and monitor therapy efficacy is in the agenda of global TB research^6–18^. The role of CD8 T-cells in the Mtb infection has been deeply studied, highlighting an important role in the recognition and killing of Mtb-infeted cells. Moreover, CD8 T-cells have an effect on Mtb replication during the active phase of TB disease and decline during the TB therapy^19^. Active TB patients have a higher frequency of Mtb specific CD8 T-cells when compared to those with LTBI and consequently, this is correlated with antigenic load^19–25^. In particular, a significant high proportion of Mtb-specific CD8 T-cells is present in smear-positive active TB patients^26^ and in subjects recently exposed to Mtb^27^. Existing interferon-γ release assay (IGRAs) are not recommended to determine treatment efficacy or for treatment monitoring in TB, either active or latent. The new generation IGRA, QuantiFERON-TB Gold Plus (QFT-Plus) contains novel antigen specific for CD8 T-cells in a second antigen tube (QFT-Plus Tube 2, TB2) designed specifically to stimulate both CD8 and CD4 T-cells. TB2 complements the first antigen tube (QFT-Plus Tube 1, TB1) containing ESAT-6 and CFP-10 peptides targeting cell-mediated immune responses from CD4 T-helper lymphocytes^28–30^.

On the base of these evidences, aim of this study is to evaluate the effect of treatment on QFT-Plus response of patients with different stages of Mtb infection, followed overtime during treatment for active TB or LTBI.

## Material and Methods

### Population characteristics

This study was approved by the Ethical Committee of “L. Spallanzani” National Institute of Infectious Diseases (INMI), approval number 72/2015. Written informed consent was obtained from all participants and/or their legal guardians, to participate in the study that was conducted at INMI. All research was performed in accordance with relevant guidelines/regulations. We longitudinally enrolled HIV-uninfected patients with pulmonary and extra-pulmonary active TB, and LTBI. Enrolled patients were classified as “confirmed TB” according the following diagnostic criteria: (i) in patients with pulmonary TB by a positive culture for Mtb from the sputum or broncholavage; (ii) in patients with extrapulmonary TB by (a) positive Mtb -specific RNA amplification (TRCReady M.TB, Tosoh, Japan) and/or Mtb -specific NAT (Home-made PCR (IS6110) GeneXpert, Cepheid; Genotype MTBDRPlus Hain Lifescience) from biological specimens or (b) by histo-pathological findings consistent with TB and presence of acid fast bacilli (AFB) in a tissue sample or (c) by positive culture for Mtb in clinical samples (pleural fluid and abscesses). Conversely, patients were classified as “clinical TB” if the diagnosis was based on clinical and radiologic criteria (having excluded other diseases) including appropriate response to standard anti-TB therapy. TB patients were enrolled within 7 days of starting the specific treatment and at the end of successfully completion of 6 month-therapy.

**Figure 1:**
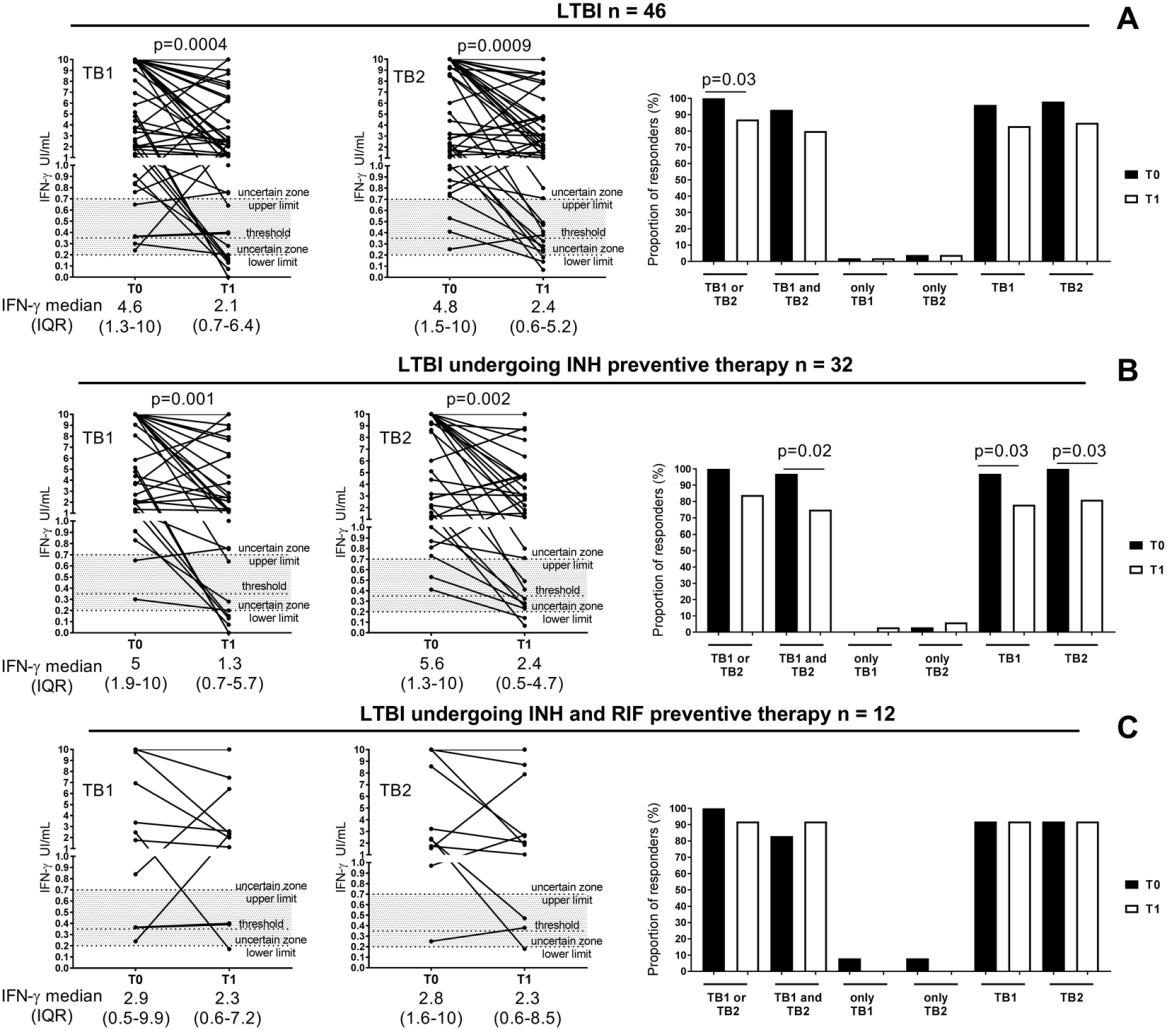
Decrease of IFN-γ response to antigens present in QFT-Plus test, TB1 and TB2, in LTBI subjects at the end of preventive treatment. (**A**) LTBI subjects, (**B**) LTBI subjects treated with INH; (**C**) LTBI subjects treated with INH and RIF. Wilcoxon signed rank test and Mc Nemar test were performed. Footnotes: IFN: interferon; IU: international unit; T0: baseline; T1: end of TB preventive therapy; threshold: cut-off according manufacturing instructions.

In the absence of clinical, microbiological and radiological signs of active TB, LTBI definition was based on a positive score to QFT-Plus (Qiagen, Hilden, Germany). LTBI subjects were enrolled before starting the specific treatment and at the end of preventive therapy completion. Preventive therapy involved isoniazid (INH) for 6 months or INH and rifampicine (RIF) for 3 months. Two patients, under different therapy regimen (2 months INH followed by 4 months RIF; 4 months RIF; respectively), have been excluded by the analysis reported in Fig. 1B,C. The LTBI group included subjects reporting a remote infection (at least three years) and subjects reporting a recent contact (no more than 3 months) with a smear-positive pulmonary TB patient. None of the subjects enrolled had previously undergone treatment with immunosuppressive drugs. Demographic and epidemiological information were collected at enrollment (Table 1).

**Table 1:** Demographic characteristic of enrolled patients.

	TB	LTBI recent	LTBI remote	Total	*p*
N (%)	28	37	9	74	
Sex female N (%)	15 (53)	15 (40)	4 (44)	34 (46)	*0.6* ^#^
Age median (IQR)	38 (28–44.7)	38 (22–52)	35 (22–61)	38 (24–50)	*0.8* ^§^
BCG-vaccinated N (%)	15 (53)	25 (67)	4 (44)	44 (59)	*0.3* ^#^
Pulmonary TB microbiologically confirmed	19 (68)	—	—	19 (68)	—
Pulmonary TB with clinical diagnosis	5 (32)	—	—	5 (32)	—
Extra-pulmonary TB with clinical diagnosis	3 (11)	—	—	3 (11)	—
Pulmonary and extra-pulmonary TB with clinical diagnosis	1 (3.6)	—	—	1 (3.6)	—
Origin (%)					*0.5* ^#^
West Europe	13 (46)	12 (32)	5 (55)	30 (40)	
East Europe	8 (29)	12 (32)	1 (11)	21 (28)	
Asian	4 (14)	8 (21)	1 (11)	13 (17)	
Africa	1 (4)	4 (10)	2 (22)	7 (9)	
South America	2 (7)	1 (3)	0 (0)	3 (4)	

Footnotes: TB: tuberculosis; LTBI: latent tuberculosis infection; BCG: Bacillus Calmette et Guérin. ^§^Kruskal Wallis test; ^#^Chi Square test.

### QFT-Plus

QFT-Plus assays was performed for each subject enrolled. QFT-Plus kits were kindly donated by Qiagen and used according to manufacturer’s instructions^30^. Levels of IFN-γ were quantified by ELISA. The results were analyzed by a QFT-Plus Analysis Software (available from www.quantiFERON.com). The software performs a quality control assessment of the assay, generates a standard curve and provides a test result for each subject. Test results were analyzed according to manufacturer’s criteria for both assays^30^. All patients resulted positive to mitogen stimulation.

### Statistical analysis

Data were analyzed using SPSS software (Version 19 FORWindows, Italy SRL, Bologna, Italy). The median and interquartile ranges (IQRs) were calculated for continuous measures. Chi square was used for categorical variables. The Kruskall Wallis test was used for comparisons among several groups and the Mann Whitney U test was used for pairwise comparisons Mc Nemar test was used for categorical matched variables. Wilcoxon signed rank test was used for pairwise comparisons.

## Results

### Population characteristics

We enrolled 74 participants: 46 LTBI subjects and 28 active TB patients. Among the active TB patients, 19 were microbiologically- and 9 clinically-diagnosed (3 with extra-pulmonary TB, 1 with pulmonary and extra-pulmonary TB, 5 with pulmonary TB). Among the LTBI subjects, 37 were recent contacts of pulmonary TB patients and 9 were remote infection. Forty percent of the enrolled subjects were from Western Europe, 28% from Eastern Europe, 17% from Asia, 9% from Africa and 4% from South America. Forty-six percent were female and 59% BCG vaccinated. We did not find any significant differences for, sex, age, BCG vaccination and origin among the different groups (Table 1).

### Effect of preventive therapy on QFT-Plus results in LTBI subjects

We evaluated the score response to antigens present in QFT-Plus test in subjects with LTBI at baseline and at the end of TB preventive therapy. LTBI subject enrollment was based on a positive QFT-Plus score at baseline. At the end of TB preventive therapy, 87% still had a positive response to either TB1 or TB2 peptides. The proportions of positive response significantly decreased at end of therapy (p = 0.03) (Fig. 1A and Supplementary Table S1). To analytically evaluate the response to the peptides contained in TB1 and TB2 tubes, we stratified the QFT Plus results according to the ability of the subjects to respond to both TB1 and TB2 peptides (“TB1 and TB2”), only to TB1 (“only TB1”) or only to TB2 (“only TB2”) (Fig. 1A and Supplementary Table S1). Among those responding to both “TB1 and TB2”, 93% responded at baseline while only 80% at the end of therapy. Moreover, the proportion of “only TB1” or “only TB2” responders did not change overtime. Among the TB1 responders, 96% responded at baseline while 83% at therapy completion. Among the TB2 responders, 98% responded at baseline while 85% at end of therapy.

### Significant decrease of IFN-γ response to antigen present in QFT-Plus test in LTBI subjects

We evaluated the results also by quantitative means (Fig. 1A). In LTBI we found that the median of TB1 response (4.6 IU/mL, IQR: 1.3–10) at baseline was significantly higher than that observed at the end of preventive therapy (2.1 IU/mL, IQR: 0.7–6.4) (p = 0.0004). Similar results were obtained in response to TB2, the median at baseline (4.8 IU/mL, IQR: 1.5–10) was significantly higher than that one at the end of treatment (2.4 IU/ mL, IQR: 0.6–5.2) (p = 0.0009). Comparing the IFN-γ response to TB1 and TB2 peptides at the same time point, we observed similar levels of IFN-γ (Supplementary Fig. S1A). Moreover we found a positive correlation between the TB1 and TB2 response both at baseline and at the end of preventive therapy (Supplementary Fig. S1B) (Baseline r = 0.92, p < 0.0001, end of TB preventive therapy: r = 0.87, p < 0.0001). To confirm that the IFN-γ decrease observed after therapy was not due to a CD8 response, we analyzed the results as “TB2 IFN-γ value-TB1 IFN-γ value”, as suggested by others^27^. However, we did not find significant differences in LTBI between baseline and end of TB preventive therapy (baseline median 0, IQR: 0.1–0.14; end of therapy median 0.04, IQR: 0.12–0.58; p = 0.7).

### Effect of the type of preventive regimen on the IFN-γ production detected by QFT-Plus in LTBI

To evaluate if the type of preventive therapy had an impact on the immunological response to antigens present in QFT-Plus test, we stratified the LTBI subjects according to preventive therapy regimen.

Among the LTBI subjects treated with INH, by inclusion criteria, 100% showed a positive response to either TB1 or TB2 peptides at baseline while at treatment completion only 84% responded (Fig. 1B and Supplementary Table S2). Stratifying the QFT-Plus results according to the ability of subjects to differently respond to TB1 and TB2 peptides, we found that 97% responded to both “TB1 and TB2” at baseline and 75% at treatment completion, and this difference was significant (p = 0.02) (Fig. 1B and Supplementary Table S2). A “TB1 only” response was found in 3% of LTBI subjects at the end of preventive therapy, whereas among the “TB2 only” responders, 3% was found at baseline and 6% at treatment completion. Among the “TB1” responders, 97% responded at baseline and 78% at therapy completion and this difference was significant (p = 0.03) (Fig. 1B Supplementary Table S2). Among the “TB2” responders, 100% responded at baseline and 81% at therapy completion and this difference was significant (p = 0.03). When the quantitative results were analyzed, (Fig. 1B) we found that the median of TB1-peptides response (5 IU/mL, IQR: 1.9–10) at baseline significantly decreased after preventive therapy (1.3 IU/mL, IQR: 0.7–5.7) (p = 0.001). Similar results were obtained in response to TB2-peptides, the median at baseline (5.6 IU/mL, IQR: 1.3–10) significantly decreased at the end of treatment (2.4 IU/mL, IQR: 0.5–4.7) (p = 0.002).

Among the LTBI subjects treated with INH and RIF, no difference was found in terms of positive score to either TB1 or TB2 at baseline and at treatment completion (100% vs 92%) (Fig. 1C and Supplementary Table 2). When the quantitative results were analyzed (Fig. 1C) we found that the median of TB1 response at baseline and after therapy completion were similar (2.9 IU/mL, IQR: 0.5–9.9 vs 2.3 IU/mL, IQR: 0.6–7.2). Similar results were obtained in response to TB2-peptides, as the median at baseline (2.8 IU/mL, IQR: 1.6–10) was comparable to that found at the end of treatment (2.3 IU/ mL, IQR: 0.6–8.5).

### Impact of the time of exposure to Mtb on the IFN-γ production detected by QFT-Plus in LTBI subjects

**Figure 2:**
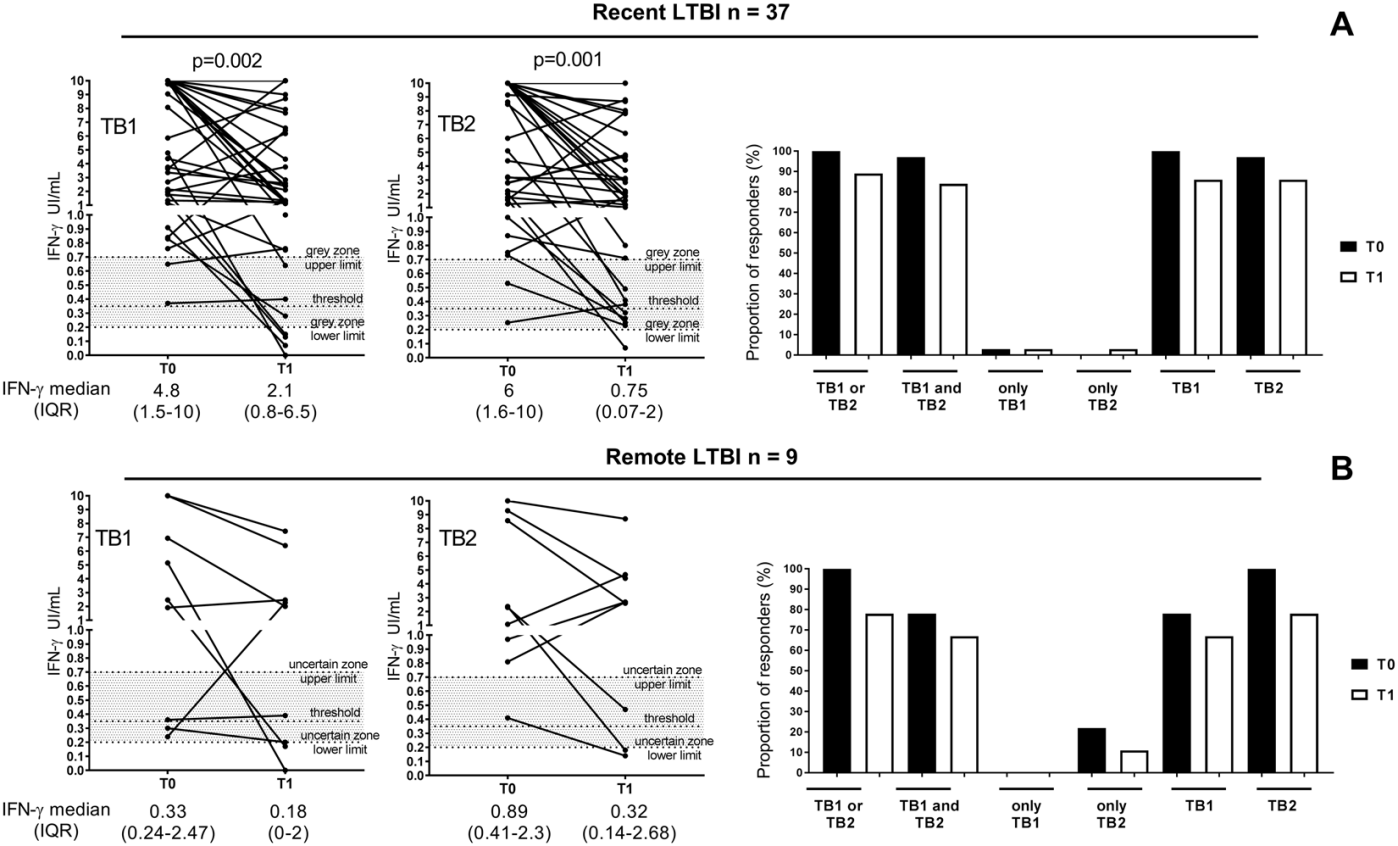
Decrease of IFN-γ response to antigens present in QFT-Plus test, TB1 and TB2, in recent LTBI subjects at the end of preventive treatment. (**A**) Recently exposed LTBI subjects, (**B**) remote exposed LTBI subjects. Wilcoxon signed rank test and Mc Nemar test were performed Footnotes: IFN: interferon; IU: international unit; T0: baseline; T1: end of TB preventive therapy; threshold: cut-off according manufacturing instructions.

To evaluate if the time of exposure to Mtb could influence the IFN-γ production we stratified the LTBI subjects according to the time of exposure to Mtb (Fig. 2 and Supplementary Table S3). Among the recent LTBI subjects, 89% showed a positive response to either TB1 or TB2 peptides at treatment completion, whereas among the remote LTBI, 78% responded at the end of treatment (Fig. 2 and Supplementary Table S3). Analyzing the quantitative results (Fig. 2), IFN-γ production decreased significantly in recent LTBI at the end of TB preventive therapy, in response to TB1 peptides (baseline: median 4.8, IQR 1.5–10; end of preventive therapy: median 2.1 IQR 0.8–6.5; p = 0.002) and TB2 (baseline: median 6, IQR 1.6–10; end of preventive therapy: median 0.75, IQR 0.07–2; p = 0.001) (Fig. 2A). No differences were observed in LTBI with a remote infection in response to TB1 peptides (IFN-γ value at baseline: median 0.33, IQR 0.24–2.47; IFN-γ value at end of preventive therapy: median 0.18, IQR 0–2) and TB2 (IFN-γ value at baseline: median 0.89, IQR 0.41–2.3; IFN-γ value at end of preventive therapy: median 0.32, IQR 0.14–2.68) (Fig. 2B). Comparing the IFN-γ production of recent and remote LTBI at the same time point (Supplementary Fig. S2), we observed at the baseline a higher IFN-γ level, although not significantly, in recent infections compared to remote infections in response to TB1 and TB2 peptides (Supplementary Fig. S2A). Differently the level of IFN-γ at the end of TB preventive treatment is similar in response to both TB1 and TB2 peptides (Supplementary Fig. S2B).

### QFT-Plus results in TB patients evaluated at baseline and at therapy completion

**Figure 3:**
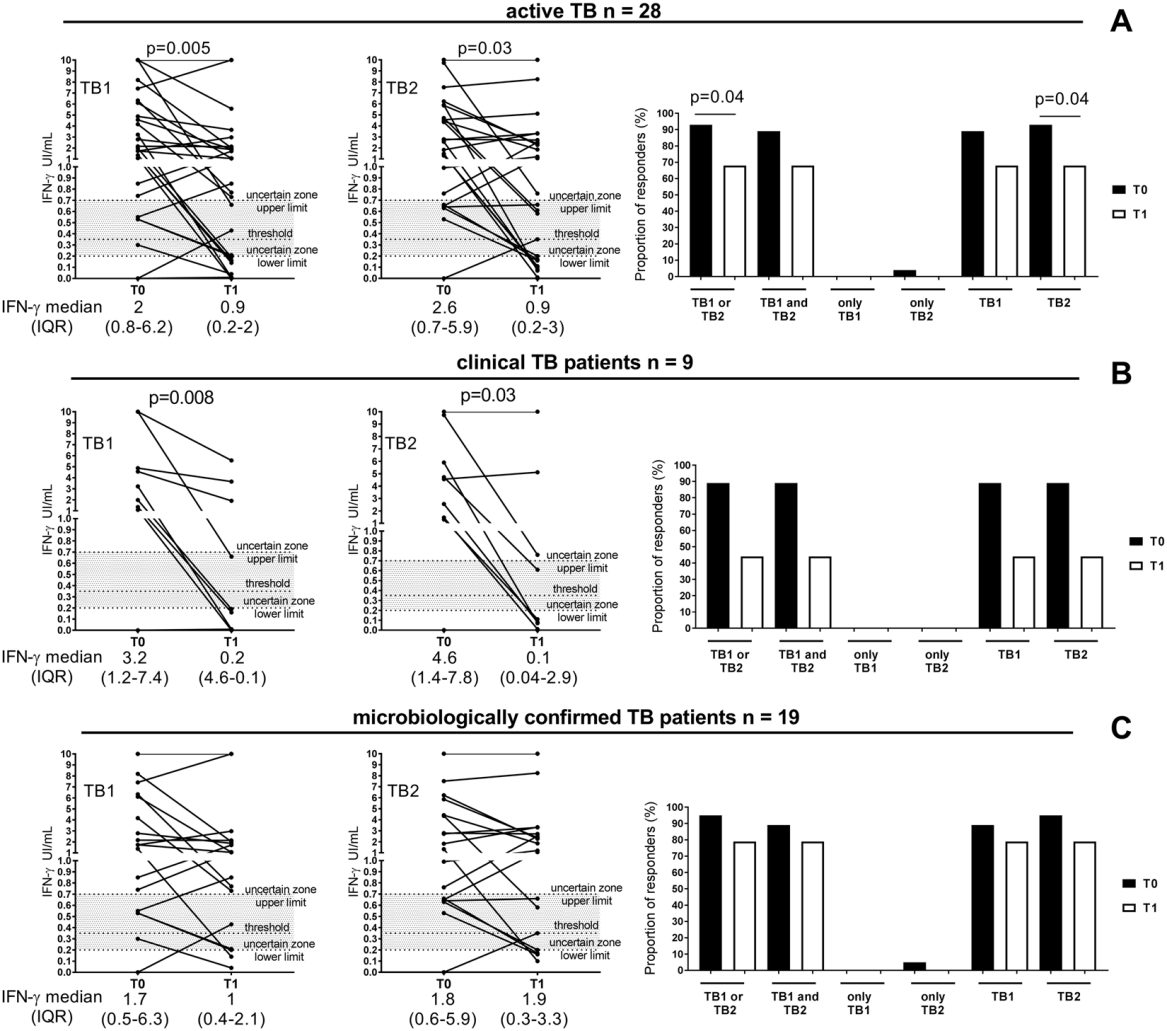
Decrease of IFN-γ response to antigens present in QFT-Plus test, TB1 and TB2, in active TB patients at the end of TB treatment. (**A**) Active TB patients, (**B**) active TB patients clinically diagnosed, (**C**) active TB patients microbiologically confirmed. Wilcoxon signed rank test and Mc Nemar test were performed. Footnotes: IFN: interferon; IU: international unit. T0: baseline; T1: end of TB therapy; threshold: cut-off according manufacturing instructions.

We also evaluated the response to antigen present in QFT-Plus test in patients with active TB. At baseline, 93% responded to either TB1 or TB2 whereas only 68% responded at treatment completion, and this difference was significant (p = 0.04) (Fig. 3A and Supplementary Table S4). Stratifying the QFT-Plus results according to the ability of subjects to differently respond to TB1 and TB2 peptides, we found that 89% of TB patients responded to both “TB1 and TB2” at baseline and 68% after therapy. We found 4% of “only TB2” responders at baseline and in nobody (0%) after therapy. TB1 responders were 89% at baseline and 68% after therapy. TB2 responders were 93% at baseline and 68% after therapy and this difference was significant (p = 0.04) (Fig. 3A and Supplementary Table S4).

### Significant decrease of IFN-γ response to antigen present in QFT-Plus test at treatment completion in TB patients

We evaluated the results also by quantitative means in TB patients (Fig. 3A). We found that the median of TB1 peptides response (2 IU/mL, IQR: 0.8–6.2) at baseline significantly decreased at therapy completion (0.9 IU/mL, IQR: 0.2–2) (p = 0.005). Similarly, the median of TB2 response at baseline (2.6 IU/mL, IQR: 0.7–5.9) significantly decreased at the end of treatment (0.9 IU/ mL, IQR: 0.2–3) (p = 0.03). Comparing the IFN-γ response to TB1 and TB2 peptides at the same time point, we found similar levels of IFN-γ (Supplementary Fig. S3A). Moreover we found a positive correlation between the TB1 and TB2 response both at baseline and at the end of preventive therapy (Supplementary Fig. S3B) (Baseline r = 0.97, p < 0.0001, end of TB therapy: r = 0.95, p < 0.0001). To confirm that the IFN-γ decrease observed after therapy was not due to a CD8 response, we analyzed the results as “TB2 IFNγ value-TB1 IFNγ value”, as suggested by others^27^. However, we did not find significant differences in active TB patients between baseline and end of therapy (baseline median 0, IQR: 0.05–0.13; end of therapy median 0.005, IQR: 0.07–0.47; p = 0.5).

### Specific TB therapy has a higher impact in decreasing IFN-γ response to antigens present in QFT Plus test in patients with clinical diagnosis compared to those with a microbiological diagnosis

To evaluate the impact of the mycobacterial load on the immunological response to antigens present in QFT-Plus test, we stratified the active TB patients according to the microbiological diagnosis. A higher proportion of responders was found in those with a microbiological diagnosis (95%) compared to those clinically diagnosed (89%) (Fig. 3B,C and Supplementary Table S5), as previously shown^29^. Interestingly, 95% of microbiologically confirmed TB patients had a positive response either to TB1 or TB2 stimulation at baseline vs 79% at treatment completion. Conversely, in clinical TB patients, 89% had a positive response at baseline vs 44% at therapy completion (Fig. 3B,C and Supplementary Table S5). Regarding the quantitative data in clinical TB patients, the therapy significantly decreased the IFN-γ production in response to TB1- (baseline: 3.2 IU/mL, IQR: 1.2–7.4; end of therapy: 0.2 IU/mL, IQR: 4.6–0.1) and TB2- peptides stimulation (baseline: 4.6 IU/mL, IQR: 1.4–7.8; end of therapy: 0.1 IU/mL, IQR: 0.04–2.9) (p = 0.008 and p = 0.03; respectively) (Fig. 3B). Differently, no significant IFN-γ value changes were observed in TB patients microbiologically confirmed (baseline: the median of TB1 peptides response: 1.7 IU/mL, IQR: 0.5–6.3; median of TB2 response: 1.8 IU/mL, IQR: 0.6–5.9) compared to end of therapy (1 IU/mL, IQR: 0.4–2.1 and 1.9 IU/mL, IQR: 0.3–3.3; respectively for TB1 and TB2) (Fig. 3C).

### Analysis of results falling in the uncertain zone (0.2–0.7 IU/mL) in LTBI and active TB patients

The positivity to the QFT-Plus assay is assigned if IFN-γ values are ≥0.35 IU/mL, as indicated by the manufacturer instruction and the CDC guidelines^30,31^. Several studies have highlight the variability of results falling close to the assay cutoff^32–34^. Considering that this assay variability is associated with discordant results upon serial evaluation, it has been suggested the use of an uncertain zone for a better interpretation of the test results^32–34^. Recently, it has been demonstrated that QFT conversions, with at least one value within the uncertain zone (0.2–0.7 IU/ml), could be partly due by experimental variability^32^.

**Figure 4:**
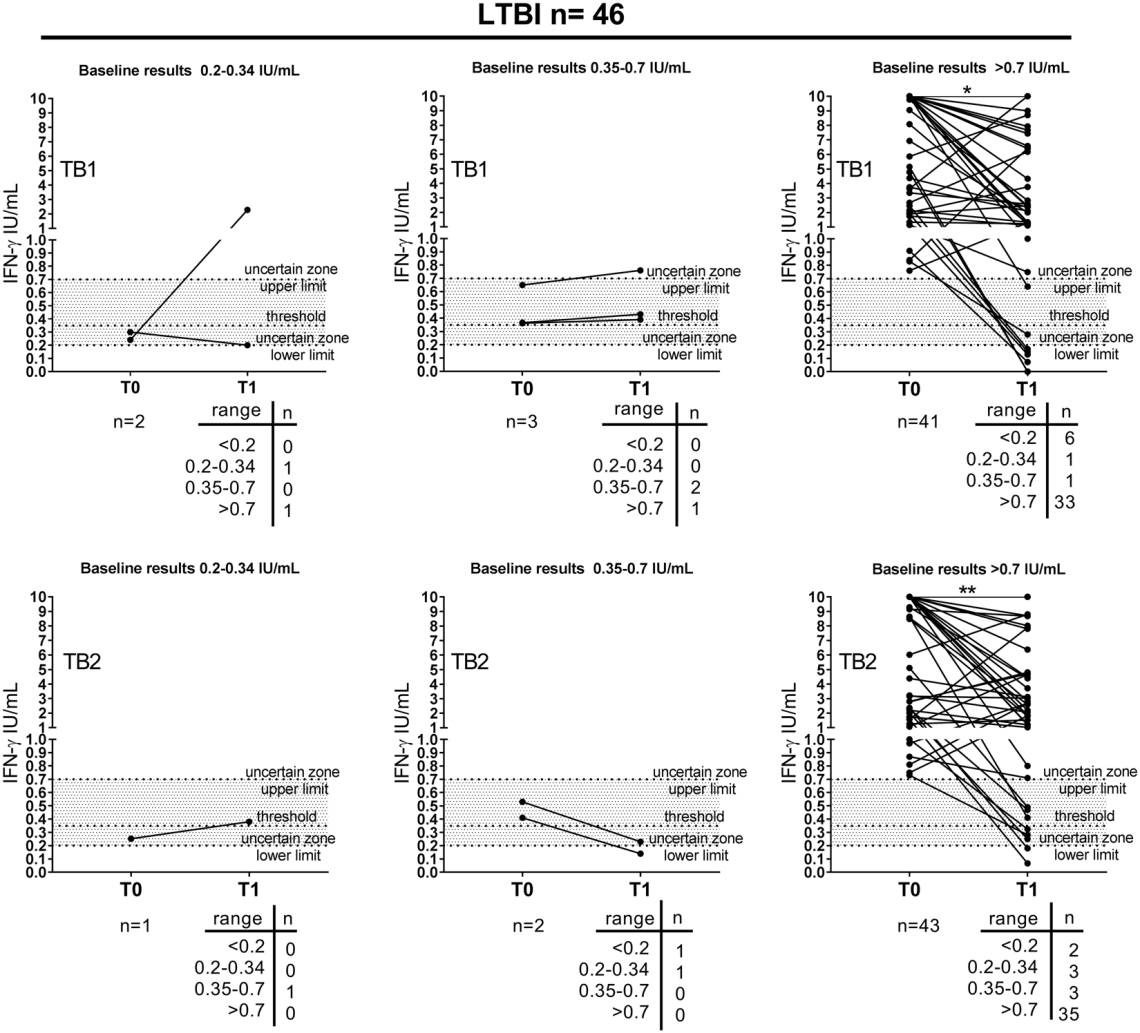
Distribution of IFN-γ values according the uncertain zone in LTBI subjects at the baseline and at the end of preventive treatment. Baseline results were stratified according the uncertain zone range. Wilcoxon signed rank test was performed to compare patients with IFN-γ values > 0.7 IU/mL at the baseline and IFN-γ values > of 0.7 IU/mL at the end of TB preventive therapy: *p = 0.003; **p = 0.01. Footnotes: IFN: interferon; IU: international unit; T0: baseline; T1: end of TB preventive therapy; threshold: cut-off according manufacturing instructions.

**Figure 5:**
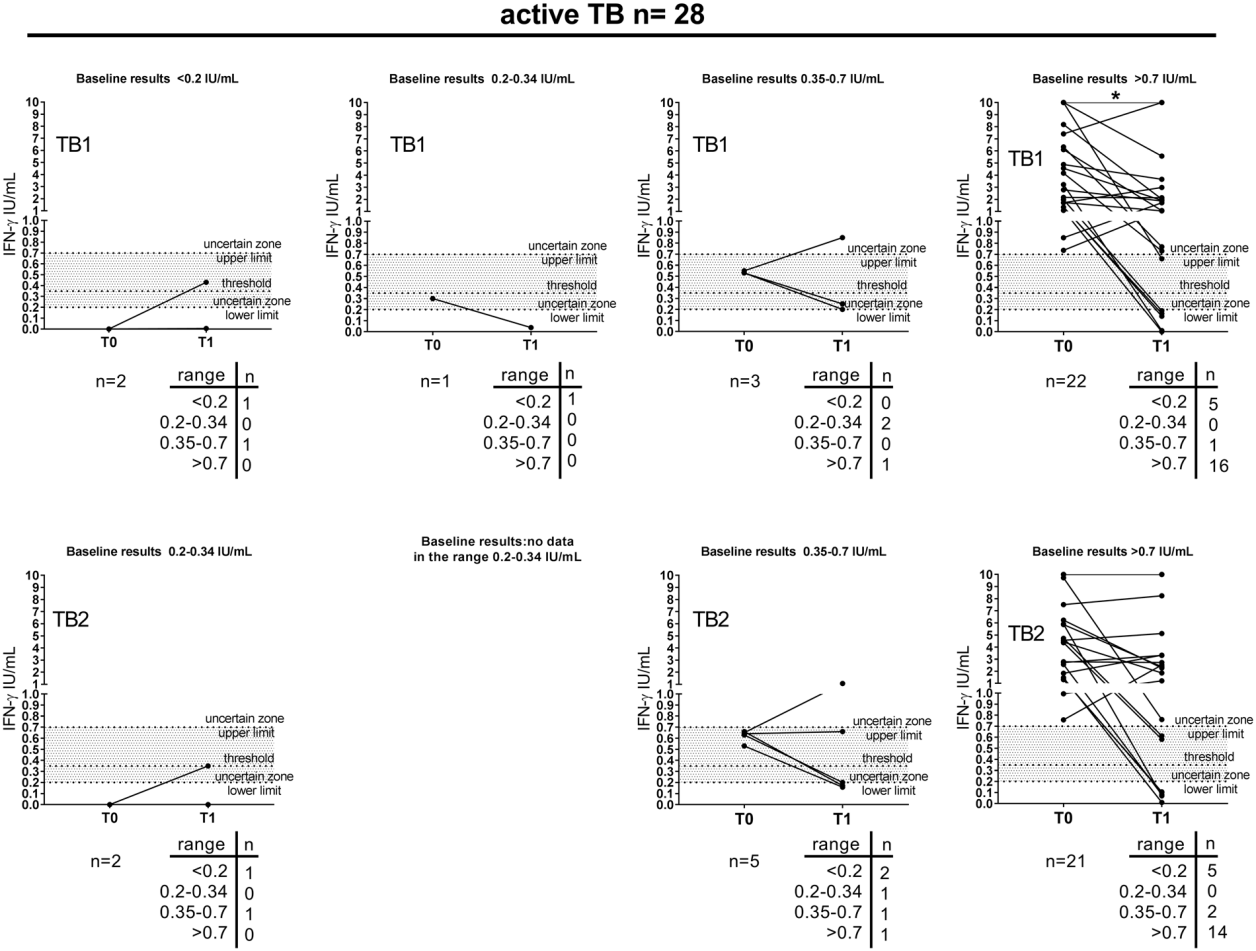
Distribution of IFN-γ values according the uncertain zone in active TB patients at the baseline and at the end of preventive treatment. Baseline results were stratified according the uncertain zone range. Wilcoxon signed rank test was performed to compare patients with IFN-γ values > 0.7 IU/mL at the baseline and IFN-γ values > of 0.7 IU/mL at the end of TB therapy, *(p = 0.04). Footnotes: IFN: interferon; IU: international unit; T0: baseline; T1: end of TB preventive therapy; threshold: cut-off according manufacturing instructions.

To evaluate the distribution of IFN-γ values in the uncertain zone, we reported our results stratifying the baseline results as follow: <0.2 IU/mL; ranging in 0.2–0.34 IU/mL; ranging in 0.35–0–7 IU/mL; >0.7 IU/mL (Figs 4 and 5). In LTBI group (Fig. 4), we found that the majority of subjects had values > 0.7 IU/mL at the baseline [89% (41/46) in response to TB1-peptides and 93% (43/46) in response to TB2-peptides). Moreover, the majority of LTBI subjects with IFN-γ values at the baseline >0.7 IU/mL had also a response >0.7 IU/mL at the end of TB preventive therapy in response to both TB1- and TB2- peptides [80% (33/41) and 81% (35/43) respectively]. Comparing only the LTBI subjects with IFN-γ values > 0.7 IU/mL (considered as values associated with “established infection”)^32^ at the baseline and at the end of therapy (33 patients for TB1; 35 patients for TB2), we confirmed a significant decrease of IFN-γ in response to TB1- (p = 0.003) and TB2-peptides stimulation (p = 0.01). A small proportion of LTBI subjects with IFN-γ values > 0.7 IU/mL had values < 0.2 IU/mL at the end of therapy, indicating a true reversion of the response to TB1- and TB2-peptides [15% (6/41) and 5% (2/43) respectively].

We also evaluated the number of reversions over time according to three different criteria: (1) “true reversion criteria” from IFN-γ values > 0.7 IU/mL to <0.2 IU/mL^32^; (2)”uncertain reversion criteria” from IFN-γ values 0.2–0.7 IU/mL to 0.2–0.7 IU/mL or to <0.2 IU/mL^32^; (3) “package insert criteria” from values ≥ 0.35 IU/mL to <0.35 IU/mL^30^ (Supplementary Table S6). In LTBI subjects, using the “true reversion criteria” we obtain a lower number of reversions (6 for TB1-peptides stimulation and 2 for TB2-peptides stimulation) compared to the results obtained using the “package insert criteria” (8 for TB1-peptides stimulation and 7 for TB2-peptides stimulation). Using the “uncertain reversion criteria”, we found only 1 reversion for TB1-peptides stimulation and 2 reversions for TB2-peptides stimulation.

In the active TB group (Fig. 5) similarly to the LTBI group, the majority of patients had a response >0.7 IU/mL at the baseline [78% (22/28) in response to TB1-peptides and 75% (21/28) in response to TB2-peptides]; and the majority of patients with IFN-γ values at the baseline >0.7 IU/mL had a response >0.7 IU/mL at the end of TB preventive therapy in response to both TB1- and TB2- peptides [73% (16/22) and 67% (14/21) respectively]. Evaluating the results of those with IFN-γ values > 0.7 IU/mL at the baseline and at end of therapy (16 patients for TB1; 14 patients for TB2), we confirmed a significant decrease of IFN-γ in response to TB1-peptides stimulation (p = 0.04).

A small proportion of active TB patients with IFN-γ values > 0.7 IU/mL had values < 0.2 IU/mL at the end of therapy, indicating a true reversion of the response to TB1- and TB2-peptides [23% (5/22) and 24% (5/21) respectively].

Using the “true reversion criteria”, we obtain a lower number of reversions (5 for TB1- and TB2-peptides stimulation) compared to that found using the “package insert criteria” (9 for TB1-and TB2-peptides stimulation) (Supplementary Table S6). Regarding the “uncertain reversion criteria”, we found only 3 reversions for TB1- and TB2-peptides stimulation (Supplementary Table S6).

Finally, we compared the distribution of IFN-γ values according the uncertain zone in response to TB1 and TB2 for each subject (Supplementary Tables S7 and S8). Interestingly, we found that the majority of LTBI subjects with a TB1-peptides response >0.7 IU/mL had also a TB2-peptides response >0.7 IU/Ml (Supplementary Table S7). Similarly, we found that majority of active TB patients with a TB1-peptides response >0.7 IU/mL had also a TB2-peptides response >0.7 IU/mL (Supplementary Table S8). Overall, these data suggest that the stimulation with TB1 and TB2 peptides induces a similar distribution of IFN-γ values.

### Quantitative values of IFN-γ in LTBI and TB subjects scored negative at the end of treatment

To further analyze the QFT-Plus results of subjects scored negative at the end of treatment, we showed the IFN-γ values expressed as IU/mL of LTBI subjects (Table 2) and active TB patients (Table 3). Among the LTBI subjects, selectively enrolled as QFT-Plus positive, we found that only one patient (Pt 2) had a positive response in the uncertain zone at the baseline and a negative response at the end of preventive TB therapy (Table 2). Among the active TB group, we found that three patient (patients 7, 17, 19) had a positive response in the uncertain zone at the baseline and a negative response at the end of TB therapy (Table 3).

**Table 2:** QFT-Plus results scored negative in LTBI at the end of TB preventive therapy; evaluation of the response to antigens present in QFT-Plus test overtime.

Patient identity	Baseline		End of preventive therapy	
	TB1 IFN-γ UI/mL	TB2 IFN-γ UI/mL	TB1 IFN-γ UI/mL	TB2 IFN-γ UI/mL
Pt 2	0.3	**0.41**	0.2	0.14
Pt 8	**1.16**	**1**	0.129	0.324
Pt 9	**0.83**	**0.73**	0.28	0.28
Pt 10	**0.91**	**1.58**	0.073	0.067
Pt 12	**1.92**	**2.01**	0.15	0.25
Pt 13	**2.47**	**2.37**	0.17	0.18

Footnotes: LTBI: Latent tuberculosis infection; TB1: tube 1. TB2: tube 2; Pt: patient; uncertain zone from 0.2 to 0.7 IU/mL.

**Table 3:** QFT-Plus results scored negative in active TB at the end of therapy; evaluation of the response to antigens present in QFT-Plus test overtime.

Patient identity	Baseline		End of therapy	
	TB1 IFN-γ UI/mL	TB2 IFN-γ UI/mL	TB1 IFN-γ UI/mL	TB2 IFN-γ UI/mL
Pt 7	0.3	**0.66**	0.038	0.171
Pt 14	0	0	0.005	0
Pt 16	**3.219**	**5.901**	0.01	0.07
Pt 17	**0.53**	**0.63**	0.2	0.2
Pt 18	**1.35**	**1.31**	0.164	0.106
Pt 19	**0.53**	**0.53**	0.207	0.158
Pt 20	**1.38**	**1.33**	0.14	0.1
Pt 21	**1.98**	**2.56**	0.19	0.08
Pt 22	**1.09**	**1.47**	0	0.01

Footnotes: TB: tuberculosis; TB1: tube 1. TB2: tube 2; Pt: patient; uncertain zone from 0.2 to 0.7 IU/mL.

## Discussion

The absence of satisfactory tools for monitoring active TB and preventive TB therapy efficacy reduces the optimal clinical management of patients^5^. Therefore, the lack of biomarker for treatment monitoring is listed among the main requirements for next generation assays, as identified among the global TB research^7^. In this study, we evaluated in a low TB endemic country such as Italy, the effect of therapy on the results obtained by the updated version of the QuantiFERON tests, the QFT-Plus, in a cohort of subjects with LTBI and active TB. We showed that the therapies decreased IFN-γ response in LTBI subjects and active TB patients leading to a significant reduction in the number of TB1- or TB2-peptides responders.

Regarding LTBI, stratifying them according to the type of preventive TB therapy used, we found a significant decrease of IFN-γ production with INH treatment but not with INH and RIF. However, the number of patients under RIF and INH treatment was lower compared to that under INH and therefore this may have reduced the possibility to show significant differences in terms of IFN-γ level modulations. According to the manufacture instructions, TB1- tube contains long peptides eliciting a CD4 T-cell response whereas TB2-tube contains, in addition to these, additional short peptides specific for the CD8 T-cells. These findings could be more refined by cytometry, demonstrating that the CD8 T-cells are mainly induced by TB2-peptides and are associated mainly to active TB^29^. If a simultaneously response “TB1 and TB2” is found, it is possible to have both a CD4 and CD8 T-cell response. The CD8 contribution may be theoretically obtained by subtracting the TB1 IFN-γ value to the IFN-γ value obtained in TB2, as done in a recent contacts setting^27,35^. Interestingly, in our study population, using this surrogate of CD8 response, we did not find significant IFN-γ values differences between responses elicited by TB2- and TB1- peptides. On the contrary, a positive correlation was observed between the TB1- and TB2- peptides responses at the baseline and at the end of therapy in both LTBI and active TB patients. However, in the recently infected subjects we reported that preventive therapy significantly decreased the response, demonstrating that the time of exposure to Mtb may influence the ability of the subjects to respond to Mtb stimulation. Probably, this is linked to the higher levels of IFN-γ values in response to TB1 and TB2 at baseline in recently infected individuals compared to remote LTBI.

Similarly, in active TB patients, the therapy significantly decreased the IFN-γ response and the number of responders. At baseline the majority of the patients displayed a “TB1 and TB2” response that decreased overtime, whereas those that at baseline had an “only TB1” or “only TB2” response revert to negative at the end of therapy. Stratifying the patients according to the microbiological status, we found that TB therapy has a higher impact in decreasing IFN-γ response to the antigens present in QFT-Plus test, in patients with clinical diagnosis compared to those with a microbiological diagnosis. Probably, the higher mycobacterial load of active TB patients microbiologically confirmed, sustains the immune response overtime. These results are in line with previous studies showing a higher Mtb specific CD4 and CD8 response in smear positive active TB patients^19,25^. An interesting study performed in Japan on active TB patients demonstrated a decline of the surrogate CD8^+^ T-cell response (difference “TB2 minus TB1”) at the end of treatment and suggested the use of QFT-Plus as a tool to help monitoring TB therapy efficacy^36^. Differently, in this study we found a decrease of IFN-γ production overtime in response to both, TB1- and TB2-peptides stimulation. Possibly, the higher TB incidence setting of Japan compared to Italy^1^ may have had an impact on the immunological status of the patients leading to a higher CD8 response compared to our study population. Based on the manufactures instructions^30^, if we found an “only TB2” response it is plausible to refer it as a CD8 T-cell response, otherwise we should have observed for the same patient also a TB1 response. Interestingly, in our study we did not observe any “only TB2” response in active TB patients at the end of TB therapy, suggesting a loss of the CD8 T-cell response in parallel with the decrease of mycobacterial load. In line with the literature reporting a correlation between mycobacterial load and CD8 T-cell response^19^, we found that patients showing an “only TB2” response had a microbiologically diagnosed TB. Therefore, investigation of the TB1- and TB2- peptides response could be a springboard to find new tools to monitor TB therapy efficacy.

Recently, deep attention has been given to the concept of the positivity threshold of QFT-Plus, defining the IFN-γ values ranging from 0.2IU/mL to 0.7/IU/mL as uncertain zone^32^. In particular, a study based on serial QFT evaluations^32^,suggests that values less than 0.2 IU/mL, are considered as “true negative values”, whereas uncertain conversions are those with at least one value within the uncertain zone (0.2–07 IU/mL) and are partially explained by technical assay variability^32^. These findings are also supported by another study from Pai et colleagues, proposing that any value of IFN-γ <0.20 IU/ml should be considered “definitely negative” and any value > 0.50 IU/mL should be considered “definitely positive”^34^. Moreover, it has been demonstrated, in a health care workers setting in a low TB incidence country, that the majority of discordant results between the old version of QuantiFERON and the new QFT Plus lies in the range of 0.2–07 UI/mL^37^. According to Moon et colleagues, the repetition of the QFT-Plus leaded to a negative score, suggesting the need of a more conservative definition of positive results in case of discordant scores^37^.

Based on these observations^32,34,37^, we have analyzed the TB1 and TB2 discordant results and the IFN-γ value distribution in the uncertain zone (>0.2 IU/mL and <0.7 IU/mL). Among LTBI subjects, one reversion was found in a subject with a discordant result ranging in the uncertain zone at the baseline and a negative response less than 0.2 IU/mL at the end of therapy. Considering that this subject was a recent contact of a pulmonary TB patient following the definition of LTBI, we believe that the first positive response was a true result and that the treatment was responsible for the lack of response at the end of therapy. In this study, it is important to note that the majority of the LTBI and active TB patients scoring negative at the end of therapy had a positive response at the baseline out of the uncertainty area^32,34^. Analyzing the distribution of all IFN-γ values over time we observed that the majority of LTBI and active TB patients had IFN-γ values > 0.7 IU/ml at the baseline and that the IFN-γ production is maintained at levels > 0.7 IU/mL in the majority of them at the end of therapy. Moreover, considering only the patients with IFN- γ values > 0.7 IU/mL at both time points, we still observed a significant decrease of IFN-γ production over time. According to previous report, repeated test with IFN-γ > 0.7 IU/mL indicate an established Mtb infection^32^. In this context, we confirm the decrease of IFN-γ values for tests at baseline and follow-up greater than 0.7 IU/mL, potentially indicating a decline of mycobacterial load during the treatment.

Using three different criteria of reversion^30,32^ to identify the non –responders, we found in both LTBI and active TB, a lower number of true reversions compared to the reversion criteria based on the manufacturing threshold. Interestingly, we found only few patients that reverted in the uncertain zone. Collectively these data suggest that it may be important to consider more stringent cut-off to identify the true reversions over time. The analysis of the distribution of IFN-γ values, in the same patient, in response to TB1- and TB2 peptides, demonstrated a similar pattern in response to both stimulations, indicating that the threshold values for TB2- and TB1-peptides stimulation could be the same.

Limitations of the study are related to the low number of patients enrolled and, among those with active TB, the lack of a comparison between patients undergoing to a successful therapy versus those that did not respond to treatment. In fact, in our study population, all active TB patients have successfully completed TB treatment. Moreover, among LTBI subjects, the impossibility to detect the bacillus makes the investigation even more difficult. Currently, we cannot consider to use the QFT-Plus as tool to monitor the treatment for LTBI and active TB. Future studies are needed to better characterize Mtb-specific response as a potential marker for monitoring TB therapy and preventive treatment effects in addition to the conventional microbiological, radiological and clinical tools.

In conclusions, we evaluated in a low TB endemic country such as Italy, the effect of therapy on QFT-Plus results in a cohort of subjects with LTBI and active TB. We demonstrated that the therapies decreased IFN-γ level in response to TB1- and TB2-peptides stimulation. Future studies are needed to characterize the real role of Mtb-specific response in monitoring the effects of TB therapy and TB preventive treatment.

## Electronic supplementary material

Supplementary figures and tables
