# Supplementary material for: Effect of therapy on Quantiferon-Plus response in patients with active and latent tuberculosis infection
Source: Sci Rep. 2018 Oct 23;8:15626. doi: 10.1038/s41598-018-33825-w (PMC6199315; doi:10.1038/s41598-018-33825-w)
Supplement: Supplementary file 1 — Supplementary figures and tables [file 41598_2018_33825_MOESM1_ESM.pdf]

Effect of therapy on Quantiferon-Plus response in patients with active and latent tuberculosis infection

Petruccioli E[1], Chiacchio T[1], Vanini V[1], Cuzzi G[1], Codecasa L[2], Ferrarese M[2], Schininà V[3], Palmieri F[3], Ippolito G[4], Goletti D[1].

[1]Translational Research Unit National Institute for Infectious Disease, L. Spallanzani, Rome; Italy,

[2] Regional TB Reference Centre, Istituto Villa Marelli, Ospedale Niguarda, Milano, Italy;

[3]Clinical Department National Institute for Infectious Disease L. Spallanzani, Rome, Italy;

[4]Scientific Direction, National Institute for Infectious Disease L. Spallanzani, Rome, Italy

Corresponding author:

Delia Goletti MD PhD

Head of the Traslational Research Unit

National Institute for Infectious Disease, L. Spallanzani,

Via Portuense 292 Rome, Italy

delia.goletti@inmi.it

tel: 0039 06 55170906

keywords:

tuberculosis; latency; diagnosis; quantiferon; quantiferon plus

Supplementary Table S1: QFT-Plus results in LTBI subjects at the baseline and at the end of preventive therapy

**QFT-Plus response to:**	**LTBI baseline (N. 46)**	**LTBI end of preventive therapy (N. 46)**	**Comparisons**
	Positive N (%)	Positive N (%)	#*p*
**either TB1 or TB2**	46 (100)	40 (87)	***0.03***
**TB1 and TB2**	43 (93)	37 (80)	*0.1*
**only TB1**	1 (2)	1 (2)	*1*
**only TB2**	2 (4)	2 (4)	*1*
**TB1**	44 (96)	38 (83)	*0.07*
**TB2**	45 (98)	39 (85)	*0.07*

Footnotes: LTBI: latent tuberculosis infection; TB1: tube 1. TB2: tube 2; N:number.# Mc Nemar test

Supplementary Table S2: QFT-Plus results in LTBI subjects according preventive therapy used

	INH			INH and RIF		
**QFT-Plus response to:**	**LTBI baseline (N. 32)**	**LTBI end of preventive therapy (N. 32)**	**Comparisons**	**LTBI baseline (N. 12)**	**LTBI end of preventive therapy (N. 12)**	**Comparisons**
	**Positive N (%)**	**Positive N (%)**	***#p***	**Positive N (%)**	**Positive N (%)**	***#p***
**either TB1 or TB2**	32 (100)	27 (84)	*0.06*	12 (100)	11 (92)	*1*
**TB1 and TB2**	31 (97)	24 (75)	***0.02***	10 (83)	11 (92)	*1*
**only TB1**	0 (0)	1 (3)	*1*	1 (8)	0 (0)	*1*
**only TB2**	1 (3)	2 (6)	*1*	1 (8)	0 (0)	*1*
**TB1**	31 (97)	25 (78)	***0.03***	11 (92)	11 (92)	*1*
**TB2**	32 (100)	26 (81)	***0.03***	11 (92)	11 (92)	*1*

Footnotes: LTBI: latent tuberculosis infection; TB1: tube 1. TB2: tube 2; N:number. INH isoniazid. RIF rifampicine; # Mc Nemar test

Supplementary table S3: QFT-Plus results in LTBI subjects according the time of exposure to *M. tuberculosis*

QFT-Plus response to:	Recent			Remote		
	LTBI baseline (N.37)	LTBI end of preventive therapy (N.37)	Comparisons	LTBI Baseline (N.9)	LTBI end of preventive therapy (N.9)	Comparisons
	Positive N (%)	Positive N (%)	#*p*	Positive N (%)	Positive N (%)	#*p*
**either TB1 or TB2**	37 (100)	33 (89)	*0.1*	9 (100)	7(78)	*0.5*
**TB1 and TB2**	36 (97)	31 (84)	*0.1*	7(78)	6 (67)	*1*
**only TB1**	1 (3)	1 (3)	*1*	0 (0)	0 (0)	*n.a.*
**only TB2**	0 (0)	1 (3)	*1*	2 (22)	1 (11)	*1*
**TB1**	37 (100)	32 (86)	*0.06*	7 (78)	6 (67)	*1*
**TB2**	36 (97)	32 (86)	*0.2*	9 (100)	7 (78)	*0.5*

Footnotes: LTBI: latent tuberculosis infection; TB1: tube 1. TB2: tube 2; N:number; # Mc Nemar test; na: not applicable because the variable are not dichotomous

Supplementary table S4: QFT-Plus results in active TB patients at the baseline and at the end of therapy

QFT-Plus response to:	Active TB baseline (N. 28)	Active TB end of therapy (N. 28)	Comparisons
	**Positive N (%)**	**Positive N (%)**	***#p***
**either TB1 or TB2**	26 (93)	19 (68)	***0.04***
**TB1 and TB2**	25 (89)	19 (68)	*0.07*
**only TB1**	0(0)	0(0)	*na*
**only TB2**	1 (4)	0(0)	*1*
**TB1**	25 (89)	19 (68)	*0.07*
**TB2**	26 (93)	19 (68)	***0.04***

Footnotes: TB: tuberculosis; TB1: tube 1. TB2: tube 2; N:number; # Mc Nemar test; na: not applicable because the variable are not dichotomous

Supplementary table S5: QFT-Plus results in active TB patients according the microbiological results at the baseline and at the end of therapy

	Active TB microbiologically confirmed			Active TB clinical diagnosis		
**QFT-Plus response to:**	**Baseline (N. 19)**	**End of therapy (N. 19)**	**Comparisons**	**Baseline (N. 9)**	**End of therapy (N. 9)**	**Comparisons**
	**Positive N (%)**	**Positive N (%)**	***#p***	**Positive N (%)**	**Positive N (%)**	***#p***
**either TB1 or TB2**	18 (95)	15 (79)	*0.4*	8 (89)	4 (44)	*0.1*
**TB1 and TB2**	17 (89)	15 (79)	*0.6*	8 (89)	4 (44)	*0.1*
**only TB1**	0 (0)	0 (0)	*na*	0 (0)	0 (0)	*na*
**only TB2**	1 (5)	0 (0)	*1*	0 (0)	0 (0)	*na*
**TB1**	17 (89)	15 (79)	*0.6*	8 (89)	4 (44)	*0.1*
**TB2**	18 (95)	15 (79)	*0.4*	8 (89)	4 (44)	*0.1*

Footnotes: TB: tuberculosis; TB1: tube 1; TB2: tube 2; N: number; # Mc Nemar test; na: not applicable because the variable are not dichotomous

Supplementary table S6: number of reversion according the definition of true reversion, uncertain reversion and manufacturing threshold.

	**True reversion** from IFN-γ values > 0.7 IU/mL to < 0.2 IU/mL<br>Number over total		**Uncertain reversion** from IFN-γ values 0.2- 0.7 IU/mL to 0.2- 0.7 IU/mL or to <0.2 IU/ mL<br>Number over total		**Reversion according the package insert threshold** from ≥0.35 IU/mL to <0.35 IU/mL<br>Number over total	
	LTBI	Active TB	LTBI	Active TB	LTBI	Active TB
TB1-peptides	6	5	1	3	8	9
TB2-peptides	2	5	2	3	7	9

Footnotes: TB: tuberculosis; TB1: tube 1; TB2: tube 2; N: number

Supplementary table S7: Similar distribution of IFN-γ values in response to peptides contained in TB1 and TB2 tubes, according the uncertain zone range in LTBI subjects.

**LTBI at the baseline**		**LTBI at the end of preventive therapy**	
**TB1-peptides stimulation range IFN-γ IU/mL (N)**	**Correspondent TB2-peptides stimulation range IFN-γ IU/mL (N)**	**TB1-peptides stimulation range IFN-γ IU/mL (N)**	**Correspondent TB2-peptides stimulation range IFN-γ IU/mL (N)**
<0.2 (0)	/	<0.2 (6)	<0.2 (2)<br>0.2-0.34 (2)<br>0.35-0.7 (1)<br>> 0.7 (1)
0.2-0.34 (2)	<0.2 (0)<br>0.2-0.34 (1)<br>0.35-0.7 (0)<br>> 0.7 (1)	0.2-0.34 (2)	<0.2 (1)<br>0.2-0.34 (1)<br>0.35-0.7 (0)<br>> 0.7 (0)
0.35-0.7 (3)	<0.2 (0)<br>0.2-0.34 (1)<br>0.35-0.7 (1)<br>> 0.7 (1)	0.35-0.7 (3)	<0.2 (0)<br>0.2-0.34 (0)<br>0.35-0.7 (3)<br>> 0.7 (0)
> 0.7 (41)	<0.2 (0)<br>0.2-0.34 (0)<br>0.35-0.7 (0)<br>> 0.7 (41)	> 0.7 (35)	<0.2 (0)<br>0.2-0.34 (1)<br>0.35-0.7 (0)<br>> 0.7 (34)

Footnotes: TB: tuberculosis; TB1: tube 1; TB2: tube 2; N: number

Supplementary table S8: Similar distribution of IFN-γ values in response to peptides contained in TB1 and TB2 tubes, according the uncertain zone range in active TB patients.

Active TB at the baseline		Active TB at the end of preventive therapy	
**TB1-peptides stimulation range IFN-γ IU/mL (N)**	**Correspondent TB2-peptides stimulation range IFN-γ IU/mL (N)**	**TB1-peptides stimulation range IFN-γ IU/mL (N)**	**Correspondent TB2-peptides stimulation range IFN-γ IU/mL (N)**
<0.2 (2)	<0.2 (2)	<0.2 (7)	<0.2 (7)
	0.2-0.34 (0)		0.2-0.34 (0)
	0.35-0.7 (0)		0.35-0.7 (0)
	> 0.7 (0)		> 0.7 (0)
0.2-0.34 (1)	<0.2 (0)	0.2-0.34 (2)	<0.2 (1)
	0.2-0.34 (0)		0.2-0.34 (1)
	0.35-0.7 (1)		0.35-0.7 (0)
	> 0.7 (0)		> 0.7 (0)
0.35-0.7 (3)	<0.2 (0)	0.35-0.7 (2)	<0.2 (0)
	0.2-0.34 (0)		0.2-0.34 (0)
	0.35-0.7 (3)		0.35-0.7 (1)
	> 0.7 (0)		> 0.7 (1)
> 0.7 (22)	<0.2 (0)	> 0.7 (17)	<0.2 (0)
	0.2-0.34 (0)		0.2-0.34 (0)
	0.35-0.7 (1)		0.35-0.7 (3)
	> 0.7 (21)		> 0.7 (14)

Footnotes: TB: tuberculosis; TB1: tube 1. TB2: tube 2; N: number

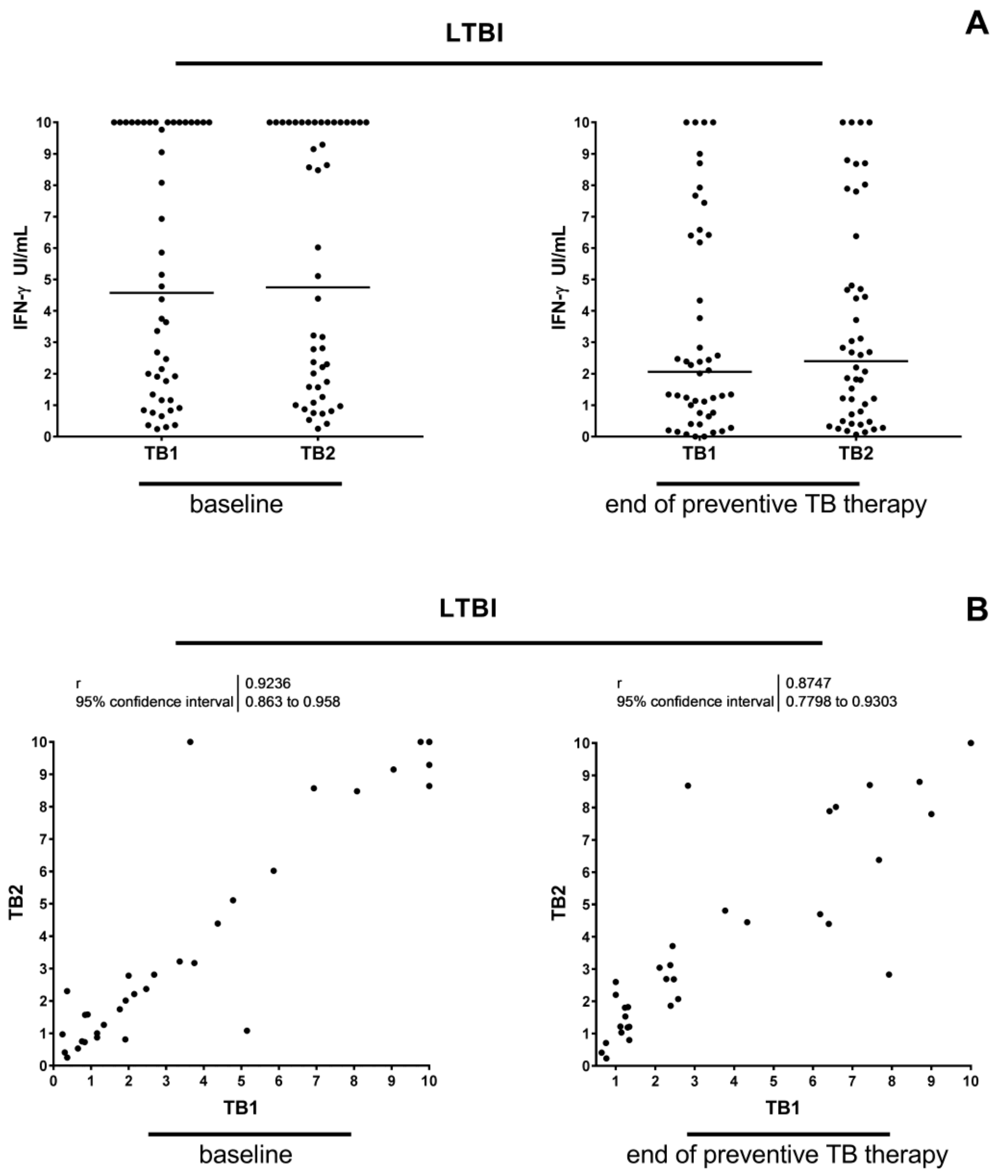

Supplementary figure S1: Comparison of IFN-γ response to antigens present in QFT-Plus test, TB1 and TB2, in LTBI subjects at the baseline and at the end of preventive therapy. A) IFN-γ response to TB1 and TB2 stimulation is similar both at baseline and at the end of preventive therapy. B) Positive correlation between the IFN-γ values in response to TB1 and TB2 stimulation both at baseline and at the end of preventive therapy. The data are presented as IU/mL. Mann–Whitney U test and Spearman correlation were performed. Footnotes: IFN: interferon; IU: international unit.

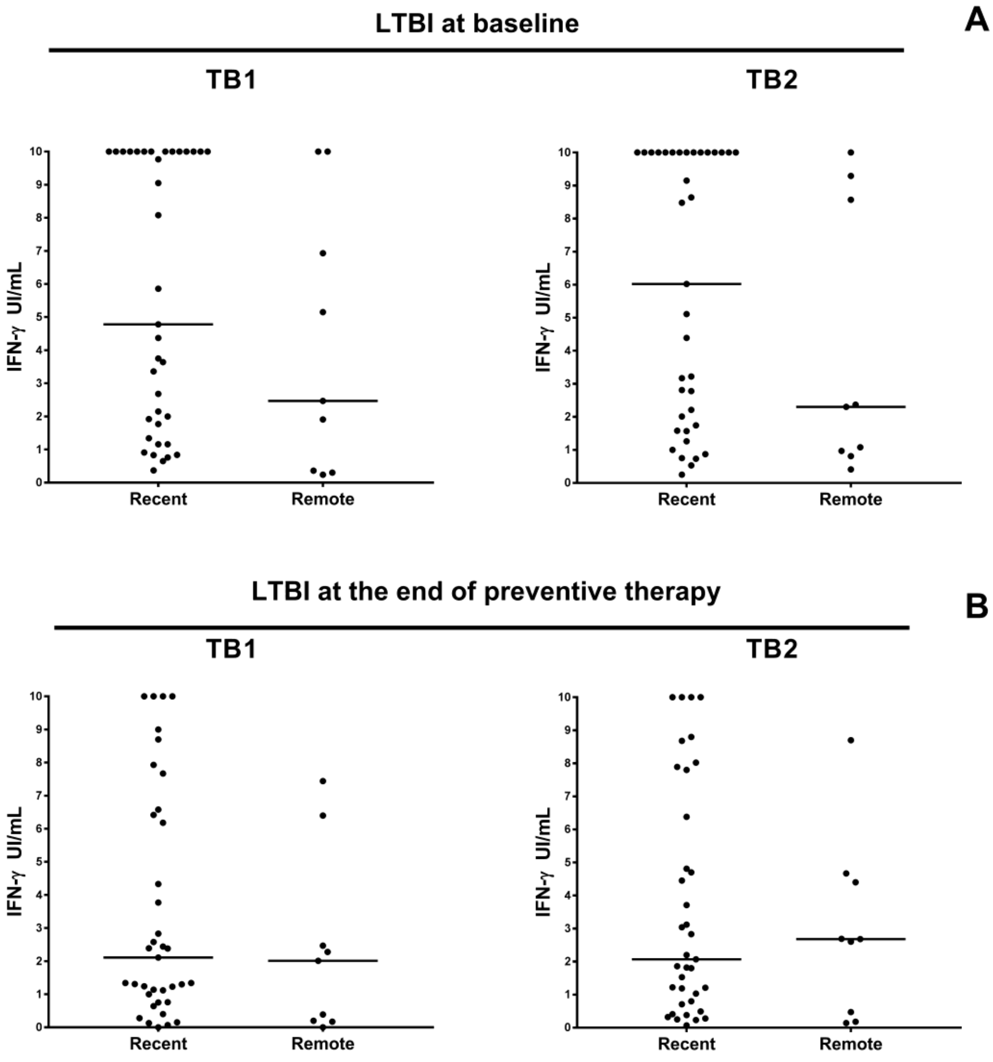

Supplementary figure S2: Comparison of IFN-γ response to QFT-Plus antigen TB1 and TB2 in recent and remote LTBI subjects at the baseline and at the end of preventive therapy. A) Recent and remote LTBI subjects at the baseline B) Recent and remote LTBI subjects at the end of preventive therapy. The data are presented as IU/mL, Mann–Whitney U test was performed. Footnotes: IFN: interferon; IU: international unit.

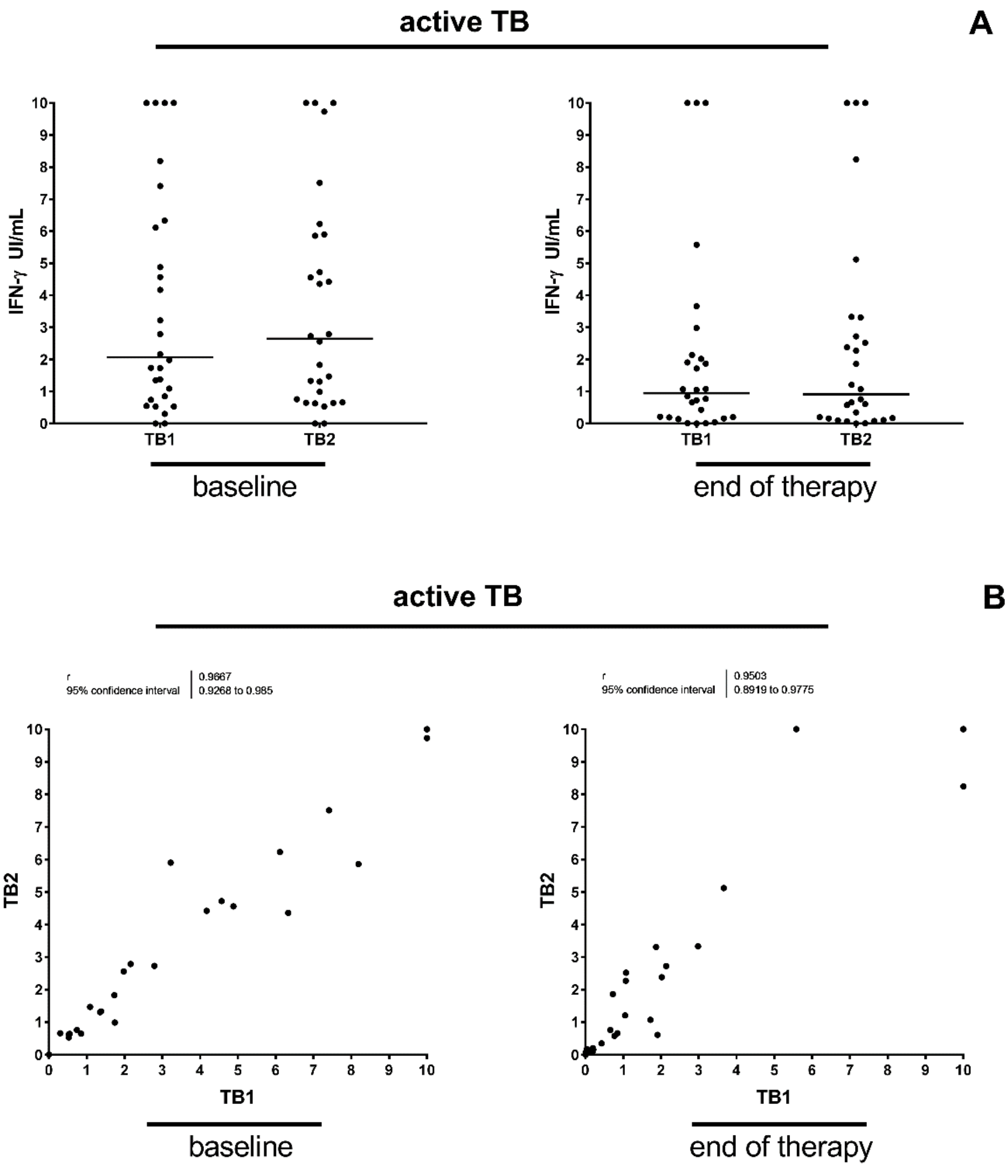

Supplementary figure S3: Comparison of IFN-γ response to QFT-Plus antigen TB1 and TB2 in active TB patients at the baseline and at the end of TB therapy. A) IFN-γ response to TB1 and TB2 stimulation is similar both at baseline and at the end of therapy; B) Positive correlation between the IFN-γ values in response to TB1 and TB2 stimulation both at baseline and at the end of therapy. The data are presented as IU/mL, Mann–Whitney U test and Spearman correlation were performed. Footnotes: IFN: interferon; IU: international unit.
